# Supplementary material for: Establishing consensus on the implementation of Anticoagulation Stewardship Program with cardiologists in Pakistan: A Delphi study
Source: PLoS One. 2025 Dec 3;20(12):e0337702. doi: 10.1371/journal.pone.0337702 (PMC12674512; doi:10.1371/journal.pone.0337702)
Supplement: S1 File — (DOCX) [file pone.0337702.s001.docx]

Supplementary File 1

**Participant**

**Information**

**Pack**

**Content**

Participant Information Sheet

Informed Consent

Informed Consent declaration

1. **PARTICIPANT INFORMATION SHEET**

This information sheet has been designed to give you information about this study. If you have any further queries, please feel free to contact to +923336565549.

**Purpose of study**

You are invited to participate in a research study designed for **“Establishing consensus on the implementation of Anticoagulant Stewardship Program with cardiologists in Pakistan: A Delphi study”.** By this study, we would like to draw the attention of members of society, government and non-governmental organizations, and other people working on patient safety.

**Confidentiality**

All information collected from your participation will be confidential and it will be used only for the research purpose. This information will be destroyed at the end of the study. Your name will be kept confidential and will not be mentioned in any report or publication. Remember, this is not a test and there are no right or wrong answers.

**B. INFORMED CONSENT**

You will be asked to sign an informed consent stating that you understand the nature of the study and what is required from you in the study.

Participation in this study is voluntarily and will not affect your privacy. You are free at any time to change your mind and withdraw from the study without needing to justify your decision.

**C. INFORMED CONSENT DECLARATION**

**“*Establishing consensus on the implementation of Anticoagulant Stewardship Program with cardiologists in Pakistan: A Delphi study*”**

**1**. I undersigned voluntarily, agree to take part in this study which I understand has been approved by responsible authorities at the Islamia University of Bahawalpur.

**2**. I confirm that a full explanation of the purpose and nature of the study has been explained to me.

**3**. I have been given the opportunity to ask questions on all aspects of the study and have understood the advice and information given as a result.

**4**. I agree to co-operate faithfully with the studying investigators with regard to my eligibility to participate in the study.

**5**. I also understand that failure to take part will no way prejudice my privacy.

**6**. I consent to the investigators having access to the information in my records, with the understanding that any publication shall not reveal my name or any other personal identifiers.

**Signature: _____________________________________________ Date: __________**

**I confirm that I have explained the nature and purpose of the study to this volunteer. If, at any time during the course of this study, new information develops that may affect the volunteer’s willingness to continue participation, a statement of this information will be provided to him/her.**

**Signature: _____________________________________________ Date: __________**

**(Researcher)**

**Witness: ______________________________________________ Date: __________**
