# Supplementary material for: Establishing consensus on the implementation of Anticoagulation Stewardship Program with cardiologists in Pakistan: A Delphi study
Source: PLoS One. 2025 Dec 3;20(12):e0337702. doi: 10.1371/journal.pone.0337702 (PMC12674512; doi:10.1371/journal.pone.0337702)
Supplement: S1 Table — (DOCX) [file pone.0337702.s002.docx]

**[Appendix Table S1](https://www.sciencedirect.com/science/article/pii/S1551741124003498#appsec1). Data Collection Tool for Cardiologists**

**Section A: Characteristics of Delphi Expert Panel**

| **Respondent ID** _____________ | |
| --- | --- |
| 1. Age ­­­­­­­­­­­­­­­­­___________ (years) | 4. Professional Experience_____________(Years) |
| 1. Gender   a) Male b) Female | 5. Position _____________ |
| 1. City 2. Lahore b) Karachi c) Islamabad   d) Rawalpindi e) Multan f) Bahawalpur  g) Rahim Yar Khan h) Sukkur | 6. Research Experience  a) Yes b) No |
| 1. Type of Hospital _____________ 2. Public b) Private c) Other |  |

**Section B: Consensus Point Regarding Anticoagulant Stewardship Program**

| **Statements** | **Agree** | **Don’t know** | | **Disagree** | |
| --- | --- | --- | --- | --- | --- |
| **Domain 1. Essential components needed to strengthen an Anticoagulant Stewardship Program.** | | | | | |
| 1. The leader or co-leaders, such as physicians and pharmacists, must be appointed for Anticoagulant Stewardship Program management and its outcomes. | 🞏 _1_ | | 🞏 _2_ | | 🞏 _3_ |
| 2. In hospitals, local anticoagulant guidelines should be developed to promote the Anticoagulant Stewardship Program. | 🞏 _1_ | | 🞏 _2_ | | 🞏 _3_ |
| 3. The hospitals should collaborate with health authorities regarding the implementation of the Anticoagulant Stewardship Program. | 🞏 _1_ | | 🞏 _2_ | | 🞏 _3_ |
| 4. Educational sessions with cardiologists in the hospitals will help to raise awareness of the Anticoagulant Stewardship Program and encourage discussion on the importance of actions regarding the appropriate anticoagulant use. | 🞏 _1_ | | 🞏 _2_ | | 🞏 _3_ |
| 5. Financial resources should be allocated for the development of anticoagulant stewardship activities. | 🞏 _1_ | | 🞏 _2_ | | 🞏 _3_ |
| 6. Comprehensive education for patients, their families, and caregivers should be essential for ensuring safe and effective management of anticoagulant therapy. | 🞏 _1_ | | 🞏 _2_ | | 🞏 _3_ |
| 7. The checklist for standardized core elements of the Anticoagulant Stewardship Program should be available in hospitals. | 🞏 _1_ | | 🞏 _2_ | | 🞏 _3_ |
| 8. The clinical experts are responsible for anticoagulant stewardship and should achieve their expertise level through advanced training. | 🞏 _1_ | | 🞏 _2_ | | 🞏 _3_ |
| 9. There must be representatives from key areas to obtain valuable perspectives from all domains of the care delivery system such as pharmacy, data analysis, and administrative leadership for the successful implementation of the Anticoagulant Stewardship Program. | 🞏 _1_ | | 🞏 _2_ | | 🞏 _3_ |
| 10. The current practices on anticoagulant management are preferable rather than the implementation of an Anticoagulant Stewardship Program by cardiologists. | 🞏 _1_ | | 🞏 _2_ | | 🞏 _3_ |
| 11. Anticoagulant guidelines must be electronically accessible to all. | 🞏 _1_ | | 🞏 _2_ | | 🞏 _3_ |
| **Domain 2. Current Status of Anticoagulant Stewardship Program in Pakistan** | | | | | |
| 12. The Anticoagulant Stewardship Program in Pakistan is in its infancy, with limited implementation across healthcare institutions. | 🞏 _1_ | | 🞏 _2_ | | 🞏 _3_ |
| 13. Some large hospitals have started implementing anticoagulant stewardship practices, but widespread adoption remains challenging. | 🞏 _1_ | | 🞏 _2_ | | 🞏 _3_ |
| 14. Collaborative efforts between pharmacists, physicians, and other healthcare providers in Pakistan are beginning to emerge as part of Anticoagulant Stewardship Program initiatives, though they are not yet widespread. | 🞏 _1_ | | 🞏 _2_ | | 🞏 _3_ |
| 15. The implementation of the Anticoagulant Stewardship Program (ASP) should be recommended in hospitals or cardiac wards. | 🞏 _1_ | | 🞏 _2_ | | 🞏 _3_ |
| 16. Patients are currently satisfied with the existing practices for managing anticoagulants, indicating that an Anticoagulant Stewardship Program may not be necessary in Pakistan at this time. | 🞏 _1_ | | 🞏 _2_ | | 🞏 _3_ |
| **Domain 3. Impact of an Anticoagulant Stewardship Program.** | | | | | |
| 17. The Anticoagulant Stewardship Program implementation will have a positive impact on the healthcare system in Pakistan. | 🞏 _1_ | | 🞏 _2_ | | 🞏 _3_ |
| 18. The successful implementation of the Anticoagulant Stewardship Program will improve patients’ health outcomes. | 🞏 _1_ | | 🞏 _2_ | | 🞏 _3_ |
| 19. The Anticoagulant Stewardship Program will promote routine INR monitoring according to patients’ indications. | 🞏 _1_ | | 🞏 _2_ | | 🞏 _3_ |
| 20. The Anticoagulant Stewardship Program will contribute to the reduction of morbidity and mortality rates associated with cardiovascular diseases. | 🞏 _1_ | | 🞏 _2_ | | 🞏 _3_ |
| 21. The Anticoagulant Stewardship Program will reduce adverse drug reactions such as severe bleeding events and thromboembolic events. | 🞏 _1_ | | 🞏 _2_ | | 🞏 _3_ |
| 22. The Anticoagulant Stewardship Program (ASP) will reduce healthcare expenditures in the healthcare system. | 🞏 _1_ | | 🞏 _2_ | | 🞏 _3_ |
| 23. The Anticoagulant Stewardship Program will provide key training and information to healthcare providers and patients, improving treatment decisions and patient adherence. | 🞏 _1_ | | 🞏 _2_ | | 🞏 _3_ |
| 24. Implementation of the Anticoagulant Stewardship Program will be a waste of resources and budget. | 🞏 _1_ | | 🞏 _2_ | | 🞏 _3_ |
| 25. The implementation of the Anticoagulant Stewardship Program may increase the workload and burden for healthcare providers. | 🞏 _1_ | | 🞏 _2_ | | 🞏 _3_ |
| **Domain 4. Constraints in the implementation of the Anticoagulant Stewardship Program.** | | | | | |
| 26. The regulatory authorities and policymakers are not focused on the implementation of an Anticoagulant Stewardship Program. | 🞏 _1_ | | 🞏 _2_ | | 🞏 _3_ |
| 27. Cardiologists are reluctant to implement the Anticoagulant Stewardship Program, as they believe it won’t lead to better outcomes. | 🞏 _1_ | | 🞏 _2_ | | 🞏 _3_ |
| 28. The inconsistent guidelines and protocols make it difficult to implement anticoagulant stewardship programs. | 🞏 _1_ | | 🞏 _2_ | | 🞏 _3_ |
| 29. Cardiologists have insufficient awareness of the Anticoagulant Stewardship Program and its benefits. | 🞏 _1_ | | 🞏 _2_ | | 🞏 _3_ |
| 30. Resistance to organizational changes, such as new procedures or policies, makes it difficult to effectively implement an anticoagulant stewardship program. | 🞏 _1_ | | 🞏 _2_ | | 🞏 _3_ |
| 31. Current training for healthcare professionals on anticoagulant management is insufficient and requires enhancement. | 🞏 _1_ | | 🞏 _2_ | | 🞏 _3_ |
| 32. Cardiologists find it impractical to hold annual meetings on anticoagulant management, as such meetings are not typically conducted, making this system difficult to implement. | 🞏 _1_ | | 🞏 _2_ | | 🞏 _3_ |
| 33. Financial limitations have a significant effect on the successful implementation of the anticoagulant stewardship program. | 🞏 _1_ | | 🞏 _2_ | | 🞏 _3_ |
