# Supplementary material for: Establishing consensus on the implementation of Anticoagulation Stewardship Program with cardiologists in Pakistan: A Delphi study
Source: PLoS One. 2025 Dec 3;20(12):e0337702. doi: 10.1371/journal.pone.0337702 (PMC12674512; doi:10.1371/journal.pone.0337702)
Supplement: S2 Table — (DOCX) [file pone.0337702.s003.docx]

**Appendix Table S2. ITEMS ELIMINATED IN THE FIRST ROUND OF THE DELPHI PROCESS**

**Round 1**

| 10 | The current practices on anticoagulants are recommended by the cardiologists, suggesting no need for an ASP program. (merged item) |
| --- | --- |
| 12 | The Anticoagulant Stewardship Program in Pakistan is in its infancy, with limited implementation across healthcare institutions. |
| 13 | Some large hospitals have started implementing anticoagulant stewardship practices, but widespread adoption remains challenging. |
| 14 | Collaborative efforts between pharmacists, physicians, and other healthcare providers in Pakistan are beginning to emerge as part of Anticoagulant Stewardship Program initiatives, though they are not yet widespread. |
| 24 | Implementation of the Anticoagulant Stewardship Program will be a waste of resources and budget. |
