# Supplementary material for: Establishing consensus on the implementation of Anticoagulation Stewardship Program with cardiologists in Pakistan: A Delphi study
Source: PLoS One. 2025 Dec 3;20(12):e0337702. doi: 10.1371/journal.pone.0337702 (PMC12674512; doi:10.1371/journal.pone.0337702)
Supplement: S3 Table — (DOCX) [file pone.0337702.s004.docx]

**Appendix Table S3. Non-consensus items carried forward to Round 2 and onwards**

**Non-consensus items included in Round 2**

| 7 | The checklist for standardized core elements of the Anticoagulant Stewardship Program should be available in hospitals. |
| --- | --- |
| 11 | Anticoagulant guidelines must be electronically accessible to all. |
| 19 | The Anticoagulant Stewardship Program will promote routine INR monitoring according to patients’ indications. |
| 25 | The implementation of the Anticoagulant Stewardship Program may increase the workload and burden for healthcare providers. |
| 27 | Cardiologists are reluctant to implement the Anticoagulant Stewardship Program, as they believe it won’t lead to better outcomes. |
| 28 | The inconsistent guidelines and protocols make it difficult to implement Anticoagulant Stewardship Programs. (merged item) |
| 29 | Cardiologists have insufficient awareness of the Anticoagulant Stewardship Program and its benefits. |
| 32 | Cardiologists find it impractical to hold annual meetings on anticoagulant management, as such meetings are not typically conducted, making this system difficult to implement. |

**The accepted and rejected items in Round 2**

| **Items accepted in Round 2** | | **Items rejected in Round 2** | |
| --- | --- | --- | --- |
| 7 | The checklist for standardized core elements of the Anticoagulant Stewardship Program should be available in hospitals. | 25 | The implementation of the Anticoagulant Stewardship Program may increase the workload and burden for healthcare providers. |
| 11 | Anticoagulant guidelines must be electronically accessible to all. | 32 | Cardiologists are reluctant to implement the Anticoagulant Stewardship Program, as they believe it won’t lead to better outcomes. |
| 19 | The Anticoagulant Stewardship Program will promote routine INR monitoring according to patients’ indications. | 27 | Cardiologists have insufficient awareness of the Anticoagulant Stewardship Program and its benefits. |
| 28 | The inconsistent guidelines and protocols make it difficult to implement Anticoagulant Stewardship Programs. (merged item) | 29 | Cardiologists find it impractical to hold annual meetings on anticoagulant management, as such meetings are not typically conducted, making this system difficult to implement. |

**Non-consensus items included in Round 3**

| 25 | The implementation of the Anticoagulant Stewardship Program may increase the workload and burden for healthcare providers. |
| --- | --- |
| 32 | Cardiologists are reluctant to implement the Anticoagulant Stewardship Program, as they believe it won’t lead to better outcomes. |
| 27 | Cardiologists have insufficient awareness of the Anticoagulant Stewardship Program and its benefits. |
| 29 | Cardiologists find it impractical to hold annual meetings on anticoagulant management, as such meetings are not typically conducted, making this system difficult to implement. |

**The accepted and rejected items in Round 3**

| **Items accepted in Round 3** | | **Items rejected in Round 3** | |
| --- | --- | --- | --- |
| 25 | The implementation of the Anticoagulant Stewardship Program may increase the workload and burden for healthcare providers. | 27 | Cardiologists have insufficient awareness of the Anticoagulant Stewardship Program and its benefits |
| 32 | Cardiologists are reluctant to implement the Anticoagulant Stewardship Program, as they believe it won’t lead to better outcomes. | 29 | Cardiologists find it impractical to hold annual meetings on anticoagulant management, as such meetings are not typically conducted, making this system difficult to implement. |
