# Supplementary material for: Establishing consensus on the implementation of Anticoagulation Stewardship Program with cardiologists in Pakistan: A Delphi study
Source: PLoS One. 2025 Dec 3;20(12):e0337702. doi: 10.1371/journal.pone.0337702 (PMC12674512; doi:10.1371/journal.pone.0337702)
Supplement: Attachment — Submitted filename: Rebuttal_R1.docx [file pone.0337702.s006.docx]

**Dear Editor**

**Subject: Submission of revised version of the manuscript**

On behalf of the research team, I would like to submit a revised version of the manuscript entitled **“Establishing consensus on the implementation of Anticoagulation Stewardship Program with cardiologists in Pakistan: A Delphi study”** for possible consideration in your esteemed journal. We would like to thank you for granting us the opportunity to submit the revised version. We are agreeable to further revise the manuscript if required.

We would also like to thank the reviewers for sparing their valuable time in the critical appraisal of our manuscript. Their constructive comments have greatly improved the quality of the manuscript.

Point-by-point answers to the reviewers’ comments are addressed and reflected in the revised manuscript in red text. A final grammar check has also been undertaken.

| **Reviewer #1** | | |
| --- | --- | --- |
|  | **Comment** | **Authors’ Response** |
|  | Kudos to this article, it represents great work. | Thanks for appreciation and positive feedback. |
|  | I am a little confused about the focus of the paper, is there more of a focus on the modified Delphi technique or the ASP?  Since I think the key element of the paper is the use of the modified Delphi technique, I think the introduction needs some background on the modified Delphi technique and whether it has been used in other similar programs. There could also be more mentions of similar use in the discussion part of the paper. | The focus of this paper was consensus development on the implementation of ASP. We used Delphi technique to achieve the study objectives. While we can add further details of Delphi methods in the introduction section, but it may be out of the scope of the Introduction section. Nevertheless, the modified Delphi technique has been explained in the methods section.  Please refer to Study Design, Page # 6. |
|  | Page 7: restructure the line "In total, there 90 cardiologist...." | It has been restructured.  Please refer to Page # 7. |
|  | Page 18: Please restructure the line: "Although this program focuses on patient health outcomes, such as reducing  adverse drug reactions (severe bleeding events and thromboembolic events), promoting routine INR monitoring according to patients’ indications, reduction of morbidity and mortality rates, being consistently identified in the literature, as well as decrease the workload and burden for healthcare providers" | The statement has been restructured. |
|  | Page 21: In the conclusion, I think it is inaccurate to state that the study identified the impact of an ASP on benefits for patients and the healthcare system; you could state that it identified a cardiology consensus on the perceived benefits for patients etc. | Amended as suggested. |
|  | Can you also list the 5 elements/items that were eliminated in the first round of the Delphi process | Attached as Appendix 2. |
|  | Finally, I wonder if the entire item list for the ASP Delphi needs to be considered a cardiologists' perception of benefits and constraints, impact, ideal implementation in the discussion since no pharmacists, other policymakers were involved in the making of the ASP and the implementation of on-the-ground policies such as these need multi-specialty collaboration and expertise. | In Pakistani healthcare system, role of pharmacist and other allied health staff in most of the hospitals is limited. Therefore, we conducted this study among those who are directly involved in patient care and those who can devise and implement policies. However, in future, if pharmacists will share this role, they could be included in such studies ahead.  This has been reflected in the Limitations.  Please refer to Page 20, Strengths and Limitations. |

On the behalf of all authors, I would again like to thank the editors and reviewers for valuable inputs and consideration.

Best regards

**Professor Muhammad Atif**

Corresponding author.
